# The Effect of ZrO_2_ Nanoparticles on the Microstructure and Properties of Sintered WC–Bronze-Based Diamond Composites

**DOI:** 10.3390/ma9050343

**Published:** 2016-05-06

**Authors:** Youhong Sun, Haidong Wu, Meng Li, Qingnan Meng, Ke Gao, Xiaoshu Lü, Baochang Liu

**Affiliations:** 1 College of Construction Engineering, Jilin University, Changchun 130000, China; syh@jlu.edu.cn (Y.S.); wuhd12@mails.jlu.edu.cn (H.W.); mli14@mails.jlu.edu.cn (M.L.); qingnanmeng@jlu.edu.cn (Q.M.); gaokenm@jlu.edu.cn (K.G.); xiaoshu.lu@aalto.fi (X.L.); 2 State Key Laboratory of Superhard Materials, Changchun 130000, China; 3 Key Laboratory of Drilling and Exploitation Technology in Complex Conditions of Minsitry of Land and Resources, Changchun 130000, China; 4 Department of Civil Engineering, Aalto University, P.O. Box 12100, Espoo FIN-02015, Finland

**Keywords:** ZrO_2_ nanoparticles, metal matrix, hot pressing, diamond impregnated composites, Orowan strengthening

## Abstract

Metal matrix-impregnated diamond composites are widely used in diamond tool manufacturing. In order to satisfy the increasing engineering requirements, researchers have paid more and more attention to enhancing conventional metal matrices by applying novel methods. In this work, ZrO_2_ nanoparticles were introduced into the WC–bronze matrix with and without diamond grits via hot pressing to improve the performance of conventional diamond composites. The effects of ZrO_2_ nanoparticles on the microstructure, density, hardness, bending strength, and wear resistance of diamond composites were investigated. The results indicated that the hardness and relative density increased, while the bending strength decreased when the content of ZrO_2_ nanoparticles increased. The grinding ratio of diamond composites increased significantly by 60% as a result of nano-ZrO_2_ addition. The enhancement mechanism was discussed. Diamond composites showed the best overall properties with the addition of 1 wt % ZrO_2_ nanoparticles, thus paving the way for further applications.

## 1. Introduction

Metal matrix-impregnated diamond composites, produced with diamond grits imbedded in a metal matrix, are widely used in diamond tools fabrication for cutting, grinding, drilling, and polishing strong materials such as rock and concrete [1,2]. The WC–bronze matrix, a composite of WC and bronze alloy (Cu-Sn-Zn-Pb), usually serves as the sintered matrix in diamond tool manufacturing [3]. Moreover, it has high strength and adjustable properties that suit different rocks, *i.e.*, that make the diamond grits easier to contact the rocks, maintaining an abrasive cutting surface. It is noted that the performances of diamond composites depend on not only the properties of the matrix and diamond materials, but also the diamond-holding ability of the matrix [4,5,6,7]. Severe service conditions (impact stresses, hydro-abrasive wear, and elevated temperature) and cost reduction demand that the mechanical properties and wear resistance be improved [8]. Several kinds of new metal matrixes have been introduced into impregnated diamond tool fabrication in recent years [9,10,11]. However, the properties of these metal matrices are difficult to adapt to different rocks and, hence, wear out much faster than diamond grits when processing hard and abrasive rocks. This leads to a short lifetime.

Therefore, it is necessary to enhance the conventional metal matrix in order to meet engineering demands. In recent years, developments of a metal matrix and alloy reinforced with particles below 100 nm offer potential and new possibilities in this regard [12,13,14,15,16,17]. By scaling down to a nanoparticle size or adding nanosized additive in metal matrices, notable improvements are expected, and the Orowan strengthening effect was found to play an important role in enhancing the metal matrix [18,19]. Moreover, nanoparticles (nanotubes) have been introduced into the metal matrix of diamond composites with novel structures in order to improve the performance of diamond tools [8,20,21].

It is known that ZrO_2_ nanoparticles (nano-ZrO_2_ hereafter) have excellent properties, namely high hardness, extreme thermal and chemical stability, and good mechanical performance. As a result, it has been widely used in reinforcing plastic, ceramic, rubber, refractory, and metals [8,22,23]. Therefore, nano-ZrO_2_ was chosen in this work for investigation of their influence on sintered WC–bronze-based diamond composites. The main aim is to seek optimal nano-ZrO_2_ content to meet the high-performance demands that is required. The microstructure, mechanical properties, wear resistance, and the related mechanism of the nano-ZrO_2_-added composites are investigated and discussed.

## 2. Materials and Methods

### 2.1. Sample Preparing

Two series of samples, including matrix samples with and without impregnated diamond, were conducted for a property test. To obtain a uniform initial WC–bronze mixture, 60 wt % WC (99.8% pure, an average particle size of 10 μm) and 40 wt % bronze (Cu 85%-Sn 6%-Zn 6%-Pb 3%, an average particle size of 48 μm) were firstly mixed in a mixer with tungsten carbide balls for 24 h at a speed of 120 rpm. Different contents of nano-ZrO_2_ (99.9% pure, average particle size of 50 nm, Beijing, China) were then mixed with the initial WC–bronze mixtures using a ball-miller for 12 h at the speed of 120 rpm. The diamond grits (20 vol % concentration, synthetic, 270–325 μm) were added into the matrix mixture for preparing diamond composite samples through three-dimensional mixing for 2 h. The mixing parameters of the initial matrix mixture and diamond composites are conventional choices [24], and that of nano-ZrO_2_ were optimized in preliminary experiments. The designation and composition of samples are given in Table 1. The mixtures were hot-pressed in graphite molds at 980 °C for 5 min. During the sintering and cooling process, a uniaxial pressure of 50 MPa was applied to the samples.

### 2.2. Characterization

The relative density of sample was determined by Archimedes’ method. The microstructure and composition were investigated by SEM (Hitachi S-4800, Tokyo, Japan) with an energy dispersive spectrometer (EDS). The phase analysis was evaluated by XRD (Shimadzu XRD6000, Kyoto, Japan). The Rockwell hardness scale C (HRC) of samples was measured by a Rockwell hardness tester (Huayin HRS-150, Yantai, China). Three-point bending strength was applied for the determination of bending strength of samples (size: 38 × 8 × 5 mm^3^).

**Figure 1:**
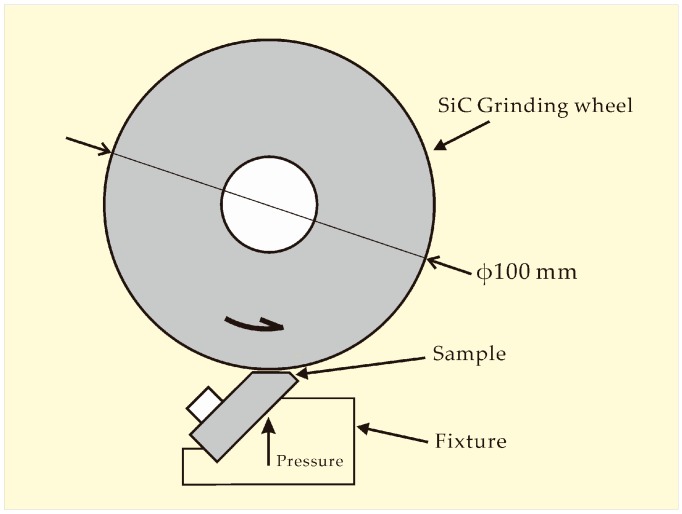
Schematic diagram of the grinding ratio test. Grinding wheels made of 80 mesh SiC grits are applied. The test parameters involved are: linear velocity 15 m/s, pressure load 500 g, and the sizes of samples range in 20 × 8 × 5 mm^3^.

In order to evaluate wear resistance of diamond composites, the grinding ratio was measured [25]. The tests were carried out on a grinding ratio measurement apparatus as illustrated in Figure 1. The grinding ratio (*R_g_*) is calculated as

(1) $$R_{g}=∆m_{g}/∆m_{S}$$

where $∆m_{g}$ and $∆m_{S}$ present the weight loss of grinding wheel and sample, respectively.

## 3. Results and Discussion

### 3.1. Compositions and Microstructures

**Figure 2:**
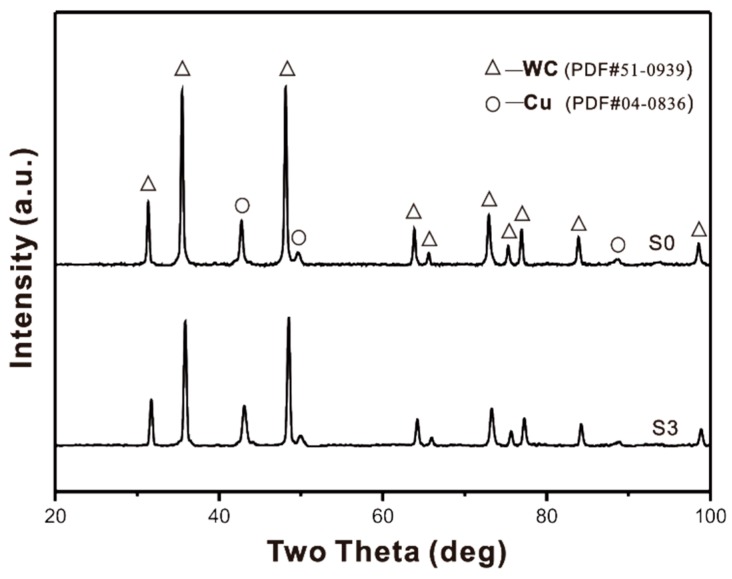
XRD patterns of samples S0 and S3.

The XRD spectra of samples S0 and S3 are shown in Figure 2. According to X-ray phase analysis data, the matrix mainly consists of two phases: WC and Cu (bronze). The XRD spectra of samples S1 and S2 are similar to those of S0 and S3. It should be noted that the introduction of nano-ZrO_2_ does not lead to a significant change in the lattice parameters of both phases according to the XRD analysis, indicating little dissolution of the hardening phase appearing in the matrix.

**Figure 3:**
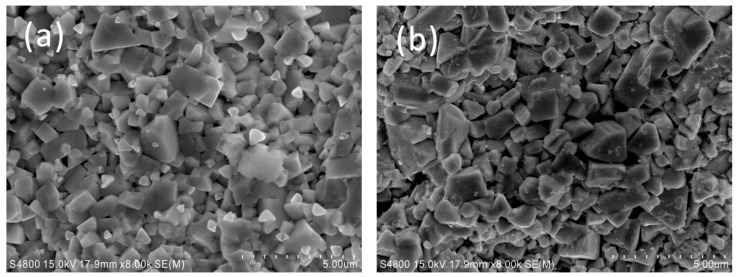
Microstructures of samples fractured faces: (**a**) S0; (**b**) S3.

**Figure 4:**
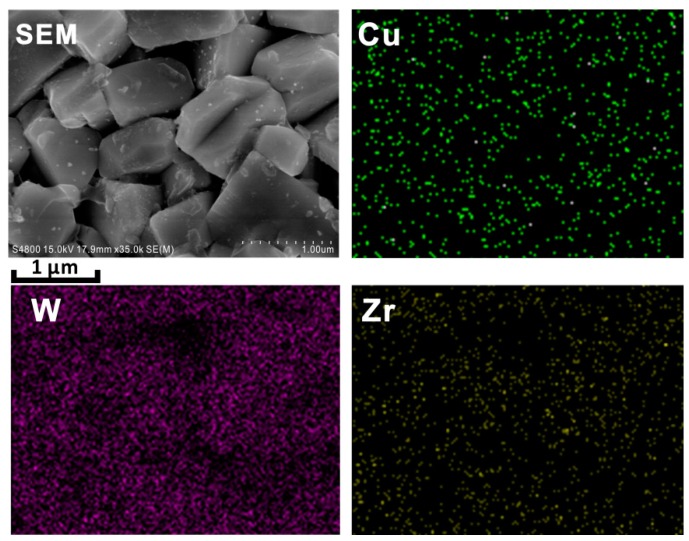
EDS element mappings of Cu, W, and Zr for the sample S3 fractured surface.

Figure 3 shows the fractured surface morphologies of samples S0 and S3. In Figure 3a,b, the 0.5–3 μm matrix grains mainly consist of rectangle-shaped grains and smooth round grains. Combined with the materials composition and EDS element mappings (Figure 4), the rectangle-shaped grains are WC, and the smooth grains are the bonding phase bronze. It is important to note that there are many nanosized particles attached to matrix grains in the sample containing nano-ZrO_2_, while the matrix grains in the sample without nano-ZrO_2_ are “clean”. Furthermore, Zr shows clear signals in EDS element mapping in Figure 4. It is therefore believed that the white nanosized particles are the added nano-ZrO_2_. It is also illustrated that the nano-ZrO_2_ is uniformly distributed in the matrix.

**Figure 5:**
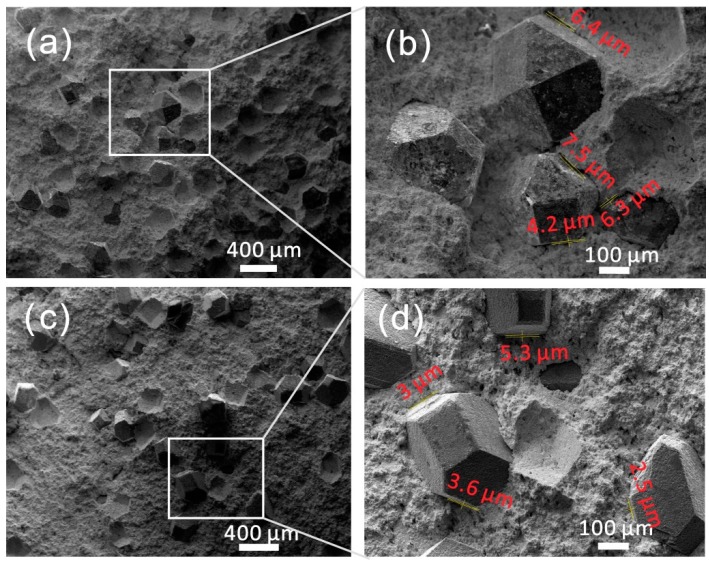
SEM images of fracture faces of samples SD0 (**a**,**b**) and SD1 (**c**,**d**). The sizes of crack width were measured in image processing software.

Fracture surface morphologies of impregnated diamond samples are illustrated in Figure 5. It shows that the diamond grits were embedded in the metal matrix. The changes in crack width in diamond/matrix interface are seen in Figure 5b,d, wherein the width decreases from 6.0 μm to 3.6 μm after adding nano-ZrO_2_. This means that the stronger metal matrix holds diamond grits more tightly due to the existence of nano-ZrO_2_ in the matrix. With a further increase in the content of the added nano-ZrO_2_, the influence of nano-ZrO_2_ content on the crack width is not significant. These structural features contribute positively to the wear resistance of impregnated diamond composites. Good diamond-holding capability of metal matrix is one of the main issues influencing the performance of the diamond tools. The holding strength at the interfaces between diamond grits and the matrix has to withstand the complex stresses developed at the individual diamond during cutting, and the diamonds should not be lost permanently by pulling out [9]. The enhancement mechanism is discussed in detail in the following section.

### 3.2. Mechanical Properties

**Figure 6:**
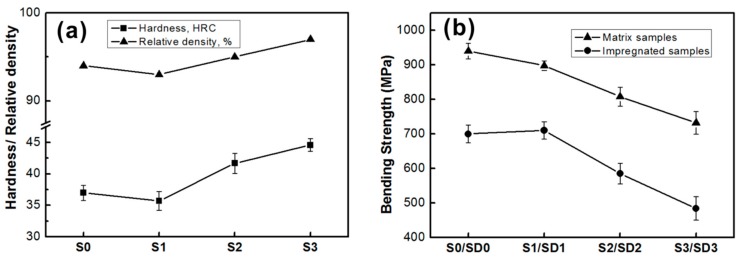
Mechanical properties of samples. (**a**) The relative density and HRC values of the matrix sample; (**b**) Bending strength of samples with and without impregnated diamond grits.

Figure 6a,b show the experimental mechanical properties of the tested samples. It can be seen that the addition of nano-ZrO_2_ results in an increase of the relative density of samples and the increased densification, which consequently enhances the hardness. For sample S3, the hardness value was improved by 20% compared with that of S0. For composites containing fine particles (<100 nm), strengthening can be attributed to the Orowan mechanism [18,19]. It has been well established that the presence of a dispersion of fine insoluble particles in a metal can considerably raise the creep resistance, due to the fact that Orowan bowing is necessary for dislocations to bypass the particles.

In addition, the mismatch in the coefficient of thermal expansion (CTE) between metal matrix (bronze > 18 × 10^−6^ K^−1^, WC ~6 × 10^−6^ K^−1^) and ZrO_2_ (~10 × 10^−6^ K^−1^) should be taken into further consideration [23,26,27]. During the cooling from the processing temperature (980 °C), thermal stresses around nano-ZrO_2_ particles cause plastic deformation stresses that reduce quickly as distance from the interface increases, this generating dislocation defects in the close vicinity of nano-ZrO_2_ [19]. The large amounts of nanoparticles are favorable for increasing the dislocation density, thus, resulting in an increase in the deformation resistance of the matrix.

The testing results also reveal that the bending strength of both matrix samples and impregnated diamond samples decreases with the increasing content of nano-ZrO_2_. Since the low wettability interfacial boundaries between ceramic ZrO_2_ and the metal matrix metal bronze are initial sources of fracture, the effective bonding area is reduced [28]. Moreover, more nanoparticles would lead to an agglomeration effect of nanoparticles and defects in the metal matrix [17], resulting in a drop in mechanical properties, especially the bending strength.

### 3.3. Wear Resistance

**Figure 7:**
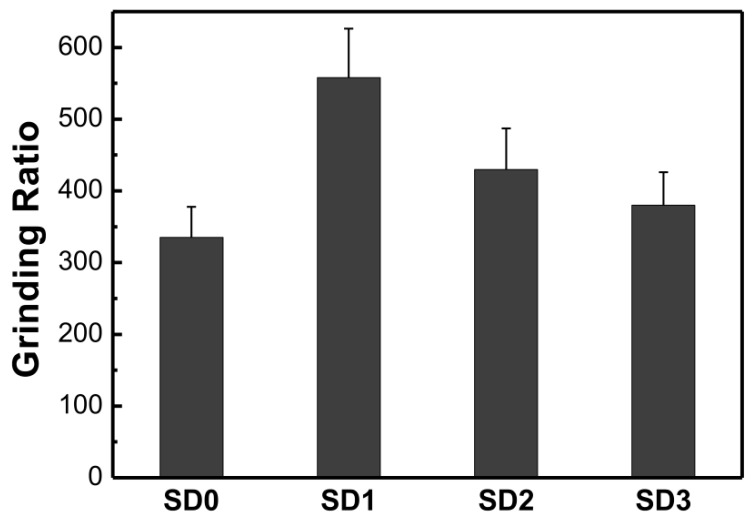
Results of grinding ratio of impregnated diamond samples.

The results for the grinding ratio of the diamond composites with different nano-ZrO_2_ amounts are shown in Figure 7. The grinding ratio is a measurement index for evaluating the wear resistance and grinding performance of diamond composites. The grinding ratio increased significantly after adding nano-ZrO_2_ particles, indicating that nano-ZrO_2_ plays an important role in wear resistance of WC–bronze diamond composites. Sample SD1 exhibits a 60% increase in the grinding ratio.

As the nano-ZrO_2_ content increases, the grinding ratio shows a decreasing trend in comparison with the highest value. The grinding ratio of the sample is dependent on the bending strength. A lower bending strength leads to a weaker support for diamond grits, meaning that diamond grits would be permanently pulled out of the metal matrix because of a severe stress during cutting. A similar relationship between bending strength and wear resistance has also been published in the literature [5,6]. Hence, the grinding ratio of samples decreases with increasing content of nano-ZrO_2_ since more nanoparticles give rise to a decrease in bending strength.

It should be noted that the grinding ratio of SD2 and SD3 is larger than that of SD0; however, the bending strength of SD2 and SD3 is smaller than that of SD0. It indicates that other factors have influence on wear resistance. As shown in Figure 6, the hardness of the matrix increases with the increase in the content of added nano-ZrO_2_, and the level of hardness of S2 and S3 is obviously larger than S0. This implies that the enhancement of the hardness of the matrix also contributes to the improvement of wear resistance. The interesting thing is that the hardness level of S1 is a little bit lower than that of S0; meanwhile, the bending strength of SD1 is similar to that of SD0. However, SD1 shows a particular increase in wear resistance. As evidenced in Figure 3, nano-ZrO_2_ particles are attached to the surface of the matrix grains, and these hard nanoparticles improve the internal friction coefficient in the matrix as well as the friction between the matrix and the diamond grit during work [10]. Diamond grits generally exhibit a rotating tendency under the cutting force, which will loosen the diamond grits. The increase of frictions helps to reduce this rotating tendency and consequently enhances the diamond retention in the matrix. Combined with an analysis of fracture faces (Figure 5), with the addition of 1 wt % nano-ZrO_2_, the width of the crack between diamond and matrix immediately decreases from 6.0 μm to 3.6 μm, meaning that the increase in diamond retention also benefits the enhancement of wear resistance.

## 4. Conclusions

The effects of ZrO_2_ nanoparticles on the microstructure, density, hardness, bending strength, and wear resistance of diamond composites were investigated. ZrO_2_ nanoparticles were found to attach to matrix grains. An increase in the concentration of nano-ZrO_2_ led to densification of the matrix by 2%–3% and a hardness increase by 20%, while the bending strength decreased by 30%. The grinding ratio of diamond composites increased significantly by 60% as a result of nano-ZrO_2_ addition. The enhancement mechanism was discussed in detail. The joint influence of hardness, bending strength, and diamond retention capability on the wear resistance of diamond composites was revealed. Composites containing 1 wt % nano-ZrO_2_ exhibited the best overall properties, thus paving the way for further research and application.

## Figures and Tables

**Table 1:** The designation, composition and mechanical properties of samples.

Samples	Composition	Relative Density (%)	Hardness (HRC)	Bending Strength (MPa)
S0	Matrix	94	37.0 ± 1.2	940 ± 23
S1	Matrix + 1 wt % nano-ZrO_2_	93	35.7 ± 1.5	898 ± 14
S2	Matrix + 2 wt % nano-ZrO_2_	95	41.7 ± 1.6	808 ± 27
S3	Matrix + 3 wt % nano-ZrO_2_	97	44.6 ± 1.0	732 ± 33
SD0	Matrix + diamond	95	-	700 ± 26
SD1	Matrix + diamond + 1 wt % nano-ZrO_2_	96	-	710 ± 25
SD2	Matrix + diamond + 2 wt % nano-ZrO_2_	96	-	585 ± 30
SD3	Matrix + diamond + 3 wt % nano-ZrO_2_	97	-	484 ± 34
